# Usefulness of a Flexible Port for Natural Orifice Transluminal Endoscopic Surgery by the Transrectal and Transvaginal Routes

**DOI:** 10.1155/2010/473080

**Published:** 2010-05-25

**Authors:** Takeshi Ohdaira, Keiichi Ikeda, Hisao Tajiri, Yoshikazu Yasuda, Makoto Hashizume

**Affiliations:** ^1^Department of Advanced Medicine and Innovative Technology, Kyshu University Hospital, 3-1-1, Maidashi, Higashi-ku, Fukuoka 812-8582, Japan; ^2^Department of Endoscopy, The Jikei University School of Medicine, Tokyo, Japan; ^3^Division of Gastroenterology and Hepatology, Department of Internal Medicine, The Jikei University School of Medicine, Tokyo, Japan; ^4^Department of General Surgery, The Jichi Medical University, Tochigi, Japan

## Abstract

We developed a flexible port for NOTES which allows the use of conventional forceps for laparoscope-assisted surgery without change. The port is not affected by the location of the through hole in the gastrointestinal tract or vagina which elicits a problem in conventional NOTES, and its length can be adjusted during surgery by cutting the port itself. The port is made of polymer resin with a low friction coefficient. Furthermore, the port walls have a square wave structure which contributes to (1) the prevention of devices, for example, endoscope, from getting stuck at the time of insertion and retrieval, (2) the prevention of port slippage in the surgical opening for port insertion, (3) the prevention of unexpected port removal, (4) the prevention of port bore deformation, and (5) the improvement of port flexibility in the longitudinal direction. We validated the insertion and retrieval capacities of commercially available forceps for laparoscope-assisted surgery and power devices. Furthermore, we used the flexible port to conduct cholecystectomy and partial gastrectomy. We could confirm that the selection of the flexible port diameter according to the device type allowed the smooth insertion and retrieval of the device and that the port produced no air leakage. We affirmed that it is possible to conduct surgery by the cross or parallel method similarly to single port surgery. We considered that the flexible port has a potential of becoming a revolutionary port in NOTES.

## 1. Introduction

### 1.1. Definition of NOTES

Natural orifice transluminal endoscopic surgery (NOTES) is a surgical procedure by which an endoscope penetrates the gastrointestinal (GI) wall through natural orifices, for example, mouth, anus, and vagina, to conduct intra-abdominal surgery.

Therefore, the positional relationship among the natural orifice, the site of GI wall penetration, and the target organ for surgery is important.

### 1.2. Current Technical Issues

Previous studies have described an attempt to perform colectomy by the ensured intra-abdominal route after setting a port for abdominal wall-penetrating laparoscope as the transrectal route [1, 2] and the use a straight port for abdominal wall-penetrating endoscope in the vaginal fornix [3]. However, the diversion of ports for laparoscope-assisted surgery has elicited the following problems: (1) shortness of port length, (2) hardness of the port, (3) and straight shape of the port. In problem (1), there are patients for whom surgery cannot be initiated because the port does not reach the site of intra-abdominal penetration in the GI tract or vagina; the port falls from the surgical opening during surgery, thus requiring a long time for reinsertion. In problem (2), the site of port insertion in the GI tract or vagina which was created to conduct NOTES is injured. Furthermore, air leakage readily occurs from the site of port insertion in the GI tract or vagina. In problem (3), a setting occurs in which surgical forceps and surgical knives do not reach the target organ for surgery because the surgical opening in the rectum or vagina and the target organ for surgery are not located on the same straight line as the port.

### 1.3. Brief Description of Improvements

We developed a port capable of exerting the following features to solve the abovementioned issues when using the conventional port and conducted an in vivo study. (1) We developed a material which allowed the adjustment of port length at will by means of a cutting device in the operating room. (2) We adopted a deformable structure for the port shape to make it fit the configuration of the rectal orifice or vagina which the port penetrates. Furthermore, we developed a sealing structure to delete air leak which occurs between the port and the device. (3) We adopted a flexible port which is freely bendable by 360 degrees and developed a structure which makes the port be adaptable to the patient's proper physique.

## 2. Materials and Methods

**Figure 1:**
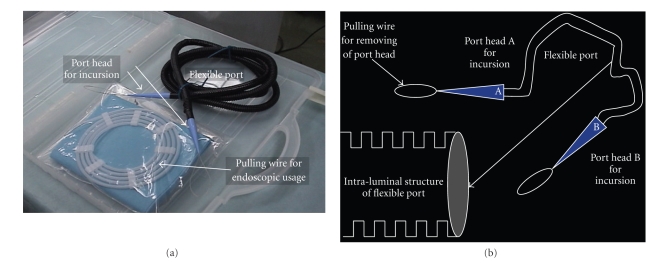
(a) A device set required to insert the flexible port. The set is composed of the flexible port itself, the guide wire for traction into the abdominal cavity, and the disposable surgical drape. First, prepare the flexible port set. One flexible port set allows the setting of two ports of the same diameter. (b) The sockets at both ends of the flexible port are made of polymer resin with a low friction coefficient. The loop wire at the socket apex is used for traction at the time of GI tract penetration and for guidance of the port-separated socket outside the body. The square wave structure of the port reduces friction and is useful in preventing the device to be inserted from getting stuck, the deformation of the port bore, and the slippage at the site of port insertion, and in improving the flexibility of the port in the longitudinal direction.

The device set required to insert the flexible port is shown in Figure 1. The set is composed of the flexible port itself, the hook wire for traction into the abdominal cavity, and the disposable surgical drape.

The features of the flexible port are listed as follows.

**Figure 2:**
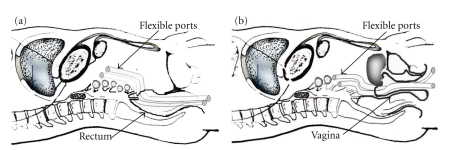
(a) Schematic layout of the flexible ports that were inserted by the transrectal route. (b) Schematic layout of the flexible ports that were inserted by the transvaginal route. The schemes of the transrectal route and the transvaginal route ensured by the flexible ports are shown in Figure 2. The flexible port is designed to be flexibly adaptable to the intra-abdominal structure and usable when inserted in the abdominal cavity by any route and in any direction. The transrectal route shown in Figure 2(a) and the transvaginal route shown in Figure 1(b) are considered when conducting the surgery of an upper abdominal organ by using the conventional forceps for laparoscope-assisted surgery without change. In the present device insertion study, we used the flexible port as shown in Figures 2(a) and 2(b). Furthermore, we conducted cholecystectomy and partial gastrectomy as shown in Figure 1(a).

1. The port material is polymer resin with a low friction coefficient.
2. The port structure adopts a square wave structure as shown in Figure 2(a).
3. The original length of the port is 1 m, and the port can be cut at will with a knife during surgery (Figure 4).
4. The port has one port-convertible socket at its each end, and the socket has a mechanism by which it can be detached by applying a given force for its removal after setting the port.
5. The conical socket for admittance has a stainless alloy wire for traction at its apex. The wire has an effect to obtain a surgical opening of 2 to 3 mm in diameter which is required for passage through the GI tract and allows traction and removal of the device outside of the body.
6. The flexible port has urethane foam bulbs for air sealing at 3 cm intervals.
7. The flexible ports prepared are 5 mm, 7 mm, 12 mm, 18 mm, and 21 mm in diameter.

The advantages generated by the above features of the flexible port are listed as follows.

1. Port polymer resin with a low friction coefficient is useful for the prevention of devices to be inserted from getting stuck.
2. The square wave structure of the port reduces friction and is useful for the prevention of devices to be inserted from getting stuck. Furthermore, the increased strength of the port in the minor axis direction can prevent the deformation of the port bore and of slippage at the site of port insertion. In addition, a marked improvement in the port's flexibility has achieved the maximal flexion angle of 110 degrees.
3. The original length of the port is 1 m, and the port can be cut at will with a knife during surgery. Namely, the flexible port provides a radical solution to the issue of anatomical individual differences.
4. The sockets at both ends of the flexible port use polymer resin with a low friction coefficient. Therefore, the port can be smoothly inserted into the abdominal cavity if there is a surgical opening of 2 to 3 mm in diameter. Furthermore, it is easy to close the surgical wound at the site of port removal because the site of port insertion is less injured.
5. The hook stainless alloy wire for traction, which is attached to the apex of the conical sockets for admittance, facilitates the passage of the device trough the GI tract and its removal outside the body by endoscopy.
6. Urethane foam bulbs for air sealing are placed at 3 cm intervals. Therefore, no air leak occurs even when cutting the port at any level.
7. The flexible ports have five different diameters, thus making them adaptable to the diameters of different devices.

### 2.1. In Vivo Study

The in vivo study was approved by the Animal Ethics Committee at the Jichi Medical University, and three farm pigs of 30 to 40 kg in body weight were used. The transrectal route was established in two male pigs, and the transvaginal route in one female pig.

The schemes of the transrectal route and the transvaginal route ensured by the flexible port are shown in Figure 2. The flexible port is designed to be flexibly adaptable to the intra-abdominal structure and to be usable when inserted into the abdominal cavity by any route and in any direction. The transrectal route shown in Figure 2(a) and the transvaginal route shown in Figure 1(b) are considered when conducting surgery of an upper abdominal organ by using conventional forceps for laparoscope-assisted surgery without change. In the present device insertion study, we used the flexible port as shown in Figure 2(a) and 2(b). Furthermore, we conducted cholecystectomy and partial gastrectomy as shown in Figure 2(a).

We used one female pig and one male pig to conduct the device insertion study with the ports of 5 mm, 7 mm, and 18 mm in diameter. The female pig was used in the transvaginal route study, and the male pig in the transrectal route study. Multiple devices were inserted and retrieved 10 times each. The presence or absence of the stuck device and the presence or absence of air leakage were examined as endpoints (Table 1).

We used two male pigs to conduct the surgical study by the transrectal route only, that is, cholecystectomy and pyloric partial gastrectomy (Table 2).

Forceps for laparoscope-assisted surgery (Karl Storz, Tuttlingen, Germany) were used as inflexible devices, and Roticulator (Covidien, MA, USA) as flexible forceps. Furthermore, Harmonic Scalpel (Johnson and Johnson, OH, USA) and LigaSure (Covidien, MA, USA) were used as incision devices. The surgical opening in the stomach was established on the anterior wall in the greater curvature of stomach. Two endoscopes for port setting and for monitoring during surgery (240i, Olympus, Tokyo, Japan) were used.

Setting of the flexible port and surgical procedures are as follows.

**Figure 3:**
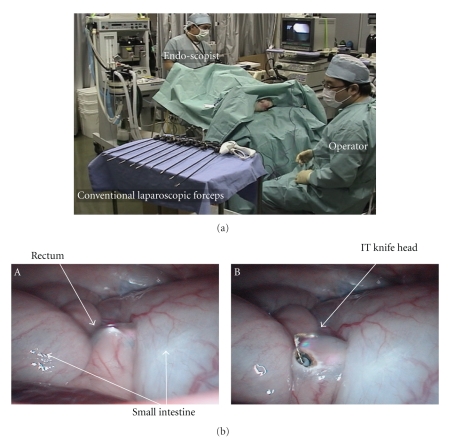
(a) Flexible port insertion procedure according to the two-surgeon method, showing the layouts of the surgeons and the forceps. (b) The surgical opening operation to insert the traction wire of the flexible port into the anterior wall of the rectum according to the two surgeon method. The safety of the procedure is ensured by an operation to be performed while monitoring the operative field with an endoscope that was inserted transgastrically.

**Figure 4:**
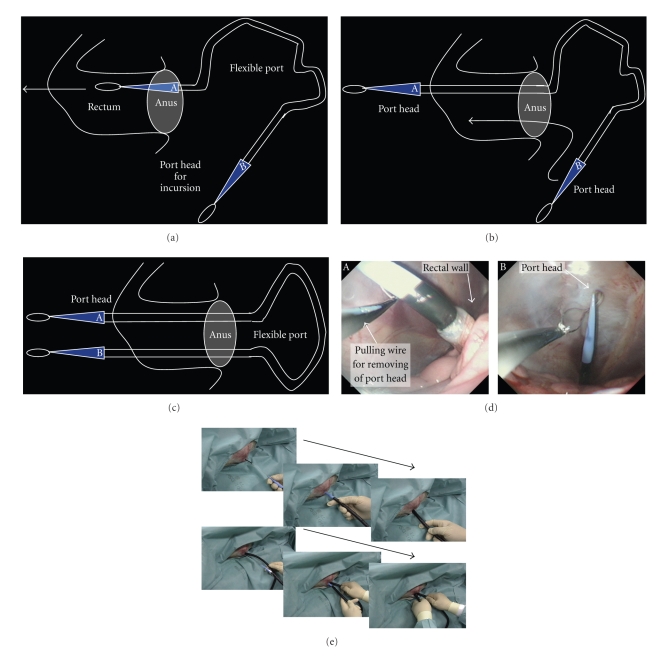
(a) Flexible port insertion procedure: the flexible port socket of one side is inserted through the surgical opening of 2 to 3 mm in diameter which was created in the rectum. This operation of creating the surgical opening of several millimeters in the rectum is performed under monitoring with the endoscope which was inserted in the abdominal cavity through the gastric wall. The flexible port is inserted into the rectal wall with the traction wire which was attached to the port insertion set through the forceps hole of the gastric camera for monitoring. (b) The flexible port socket of the other side is inserted according to the same procedure as the method in Figure 3(a). Insertion is assisted with the traction wire which is inserted into the abdominal cavity through the forceps hole of the gastric camera for monitoring. (c) The setting of two flexible ports in one set through the operations described in Figures 4(a) and 4(b) is completed. At this moment, make sure to adjust the port length which is required for the abdominal cavity. (d) The photographs of the abdominal cavity monitoring in operations mentioned in Figures 4(a)–4(c): (1) the insertion socket with the loop wire, which is placed to the apex of the flexible port immediately after penetration through the rectal wall. (2) The scene indicates traction with the traction wire which was inserted into the abdominal cavity via the forceps hole of the endoscope through which the socket with the loop wire attached to the apex of the flexible port was guided into the abdominal cavity transgastrically. By this operation, the flexible port is inserted from the rectal wall to the abdominal cavity for a required length and is placed. (e) Photographs ex vivo in Figures 4(a)–4(c). Method of flexible port insertion: the flexible port is inserted one by one. The flexible port socket with the wire loop for traction is pulled toward the mouth with the traction wire which was drawn from the anus via the sheath that had been inserted through the forceps hole of the transgastric endoscope and is guided to the abdominal cavity.

**Figure 5:**
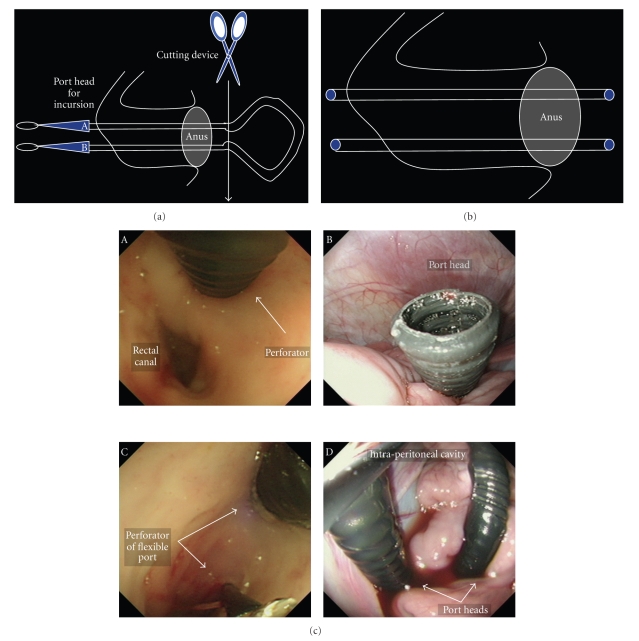
(a) Method to adjust flexible port length outside the body: after completing the setting of the flexible port to the rectal wall, the portion of the port which is exposed outside the body is cut at an arbitrary level. The original length of the port is 1 m. This separation operation to adjust port length can be conducted easily during surgery while checking required length. (b) Two flexible ports of the same diameter can be set in one insertion. In the case of changing the port, the setting can be made by adding the flexible ports of different diameters. (c) Flexible ports whose setting was completed in Figures 4(a) and 4(b). (1) A flexible port that was completely set to the anterior wall of the rectum. (2) Intra-abdominal picture of the first flexible port whose setting was completed. (3) The second flexible port of 5 mm in diameter was inserted. (4) Intra-abdominal picture of the second flexible port whose setting was also completed.

**Figure 6:**
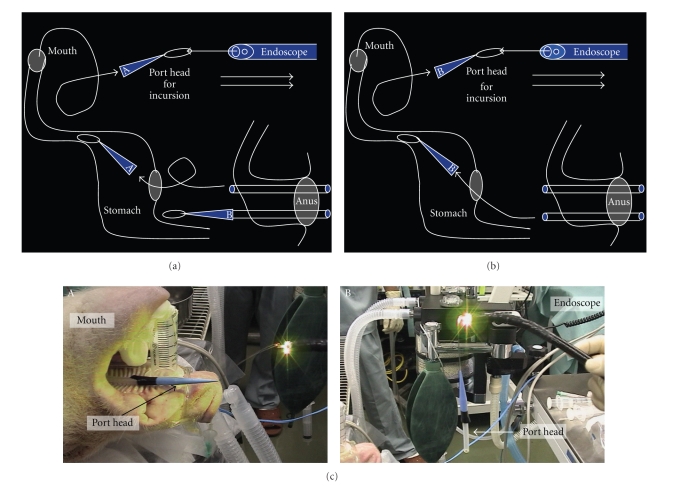
(a) The method to carry the apex socket for flexible port insertion out of the body after setting the flexible port: after completing the setting of the flexible port in the abdominal cavity, the apex socket for flexible port insertion is carried out of the body. The carriage is performed by grasping the wire attached to the socket apex with the traction wire which is attached to the flexible port set. The traction wire is inserted into the abdominal cavity after passage through the forceps hole of the transgastric endoscope. The removal of the apex socket from the flexible port is achieved with an instant slight force and by traction for a short distance. The socket separated from the port is carried outside the body from the mouth after passage through the gastric cavity and esophagus. (b) It is possible to set a given number of ports and to carry the sockets outside the body by repeating the procedures as those in Figure 5(a). (c) The sockets for port insertion that were carried outside the body in Figures 5(a) and 5(b): (a) indicates a scene in which the sockets for guidance of the flexible port with the loop wire are removed from the mouth. (b) indicates the sockets for guidance of the flexible ports with the loop wire which were retrieved completely.

**Figure 7:**
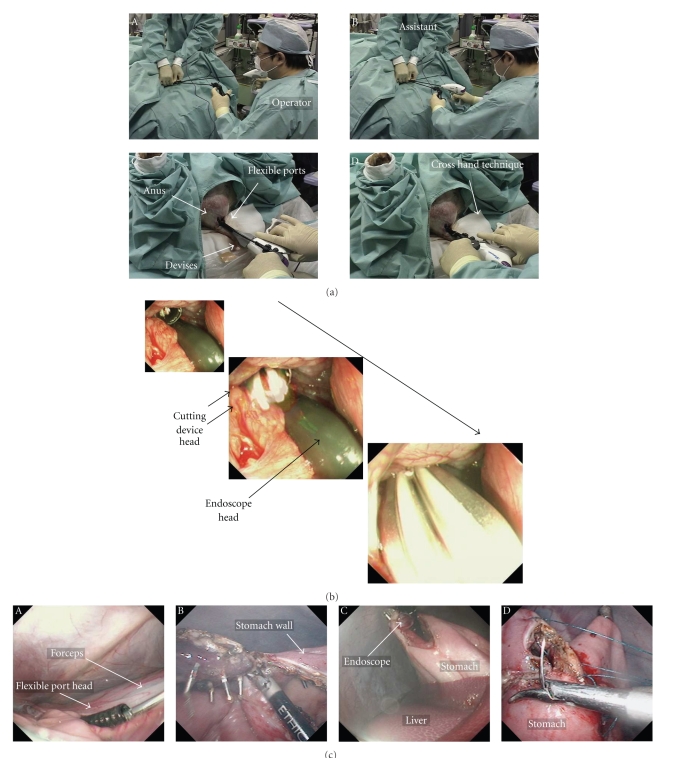
(a) A scene in which the device for conventional laparoscope-assisted surgery is used via the flexible port: a scene, in which 5 mm LigaSure and 5 mm grasping forceps are inserted from the anus into the abdominal cavity and are used, is shown (A) and (B). It is possible to conduct surgery according to the cross and parallel methods (C) and (D) similarly to SPS. (b) Single arrow: the apex of the 10 mm endoscope was inserted through the flexible port of 12 mm in diameter. The double arrow: the head of LigaSure which was inserted through the flexible port of 12 mm in diameter. (c) Full-thickness gastrectomy which was conducted with the flexible port: (A) the head of LigaSure of 10 mm in diameter which was inserted into the abdominal cavity through the flexible port. LigaSure of 10 mm in diameter made it possible to treat the great omentum and mobilize the stomach. (B) Full-thickness gastrectomy using Harmonic Scalpel: it was necessary to pull the stomach toward the tail with 45 cm grasping forceps in order to conduct full-thickness gastrectomy. (C) A scene indicating the completion of full-thickness partial resection of the stomach dummy lesion.(D) Closure of the gastric incision with a needle holder: when inserting a needle into the abdominal cavity through the flexible port, an ordinary needle is curved in the form of ski before use. A magnetic traction device developed by Ohdaira is used as the auxiliary device for resection.

**Figure 8:**
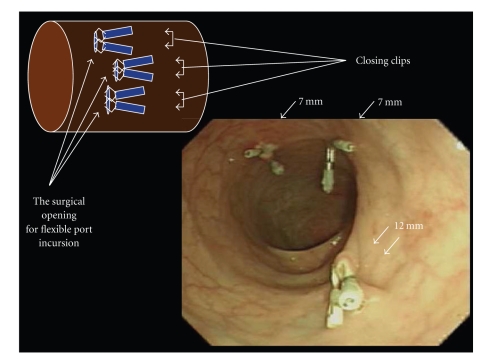
An endoscopic picture of the rectal wall in which the surgical opening for the flexible port was closed with a hemostatic clip. Two ports of 7 mm in diameter and one port of 12 mm in diameter were used.

1. Setting of the camera for abdominal monitoring: the upper endoscope is used as the conventional laparoscope according to the two-surgeon method, and the intra-abdominal insertion of the flexible port is assisted. The layouts of the surgeons and the forceps are shown. First, the endoscope is orally inserted into the stomach. Subsequently, the hook knife (Olympus, Tokyo, Japan) is used to create a surgical opening of 5 mm in diameter on the anterior wall of the greater curvature of stomach, followed by the intra-abdominal insertion of the endoscope. The above procedures allow the monitoring of surgical procedures in the abdominal cavity (Figure 3(a)).
2. Creation of a surgical opening to insert the traction wire for the flexible port on the anterior wall of the rectum according to the two-surgeon method: an ultrasonic endoscope for laparoscope-assisted surgery is used to identify the site of peritoneal reflection on the anterior wall of the rectum, and the hook knife is used to create a surgical opening of 2 to 3 mm in diameter sufficient for passage of the wire at a distance 3 cm or more away toward the mouth. All these operations are performed under monitoring with a transgastric endoscope (Figure 3(b)).
3. Method to insert the flexible port: the flexible port socket of one side is inserted through a surgical opening of 2 to 3 mm in diameter which was created in the rectum. This operation of creating the surgical opening is performed under monitoring with an endoscope which was inserted in the abdominal cavity through the gastric wall. The flexible port is inserted into the rectal wall by using the traction wire attached to the port insertion set through the forceps hole of a gastric camera for monitoring (Figure 4(a)).
4. The flexible port socket of the other side is inserted according to the same procedures as those described in Figure 3(a). Insertion is assisted by using the traction wire which is similarly inserted into the abdominal cavity through the forceps hole of the gastric camera (Figure 4(a)).
5. The setting of two flexible ports in one set through the operations described in Figures 4(a) and 4(b) is completed. At this moment, make sure to adjust port length which is required for the abdominal cavity (Figure 4(c)).
6. The photographs of the abdominal cavity monitoring in operations described in Figure 4(a) to Figure 4(c) are shown in Figure 4(d). (1) The insertion socket with the loop wire, which is placed to the apex of the flexible port immediately after penetration through the rectal wall, is shown. (2) The scene indicates traction with the traction wire which was inserted into the abdominal cavity via the forceps hole of the endoscope through which the socket with the loop wire attached to the apex of the flexible port was guided into the abdominal cavity transgastrically. By this operation, the flexible port is inserted from the rectal wall to the abdominal cavity for a required length and is placed.
7. Photographs ex vivo in the operations described in Figure 4(a) to Figure 4(c) are shown in Figure 4(e). Method of flexible port insertion: the flexible port is inserted one by one. The flexible port apex socket with the wire loop for traction is pulled toward the mouth with the traction wire which was drawn out of the anus via the sheath that had been inserted through the forceps hole of the transgastric endoscope and is guided into the abdominal cavity.
8. Method to adjust flexible port length outside the body: after completing the setting of the flexible port to the rectal wall, the portion of the port which is exposed outside the body is cut at an arbitrary level. The original length of the port is 1 m. This separation operation to adjust port length can be conducted easily during surgery while checking required length (Figure 5(a)).
9. Two flexible ports of the same diameter can be set in one insertion. In the case of changing the port, the setting can be made by adding flexible ports of different diameters (Figure 5(b)).
10. Monitoring photographs of the sites of first and second port insertions with a colonoscope which was inserted in the rectum is shown in Figure 5(c)-A and C. The gastroscopic pictures of two ports whose insertion in the abdominal cavity was completed are shown in Figure 4(c)-B and D. The colonoscopic photographs in Figure 4(c)-A and C show the tightness of the site of port admittance to prevent air leakage.
11. The method to carry the apex socket for flexible port insertion out of the body after setting the flexible port is shown in Figure 6(a). After completing the setting of the flexible port in the abdominal cavity, the apex socket for flexible port insertion is carried out of the body. The carriage is performed by grasping the wire attached to the socket apex with the traction wire which is attached to the flexible port set. The traction wire is inserted into the abdominal cavity after passage through the forceps hole of the transgastric endoscope. The removal of the apex socket from the flexible port is achieved with an instant slight force and by traction for a short distance. The socket separated from the port is carried outside the body from the mouth after passage through the gastric cavity and esophagus.
12. It is possible to set a given number of ports and to carry the sockets outside the body by repeating the procedures described in Figures 6(a) and 6(b).
13. The sockets for port insertion which were carried outside the body in Figure 1 and Figure 2 are shown in Figure 6(c). (a) indicates a scene in which the sockets for guidance of the flexible port with the loop wire are removed from the mouth. (b) indicates the sockets for guidance of the flexible ports with the loop wire which were retrieved completely.
14. Figure 7(a) indicates a scene in which the device for conventional laparoscope-assisted surgery is used via the flexible port. A scene, in which 5-mm LigaSure and 5-mm grasping forceps are inserted from the anus into the abdominal cavity and are used, is shown (A and B). It is possible to conduct surgery according to the cross and parallel methods (C and D) similarly to single port surgery (SPS).
15. The endoscope and the incision device, which are inserted into the abdominal cavity through the flexible port, are shown in Figure 7(b). The single arrow indicates the apex of the 10-mm endoscope which was inserted through the flexible port of 12 mm in diameter. The double arrow indicates the head of LigaSure which was inserted through the flexible port of 12 mm in diameter.
16. The reality of full-thickness gastrectomy, which was conducted with the flexible port, is shown in Figure 7(c). The head of LigaSure of 10 mm in diameter which was inserted in the abdominal cavity through the flexible port. It was possible to treat the great omentum and to mobilize the stomach by using LigaSure of 10 mm in diameter (Figure 7(c)-A). Full-thickness gastrectomy using Harmonic Scalpel: it was necessary to pull the stomach toward the tail with the 45 cm grasping forceps in order to conduct full-thickness gastrectomy (Figure 7(c)-B). A scene indicating the completion of full-thickness partial resection of the stomach dummy lesion is shown in Figure 7(c)-C. Closure of the gastric incision with a needle holder: when inserting a needle into the abdominal cavity through the flexible port, an ordinary needle is curved in the form of ski before use. A magnetic traction device developed by Ohdaira is used as the auxiliary device for resection (Figure 7(c)-D).
17. The flexible port is removed after completing the surgical procedures.
18. Close the site of flexible port admittance which enlarged to approximately 7 mm in diameter. In fact, the insertion hole after the removal of the flexible port shrinks to a diameter smaller than the diameter of the inserted port. Therefore, it is possible to use a skin stapler and an hemostatic clip for endoscope. The endoscopic picture of the rectal wall where the surgical openings for ports are closed with hemostatic clips is shown in Figure 8. Two ports of 7 mm in diameter and one port of 12 mm in diameter were used.

## 3. Results

### 3.1. In Vivo Study

The results of the port insertion study are shown in Table 1. Furthermore, the results of cholecystectomy and pyloric partial gastrectomy in pigs are shown in Table 2.

Injury and deformation of the port insertion hole: after surgery, any deformation or enlargement did not occur at all to the bore of the site of flexible port setting in the rectum and vagina.

Requisite for the length of the device used: conventional forceps for laparoscope-assisted surgery were required to have a length of 35 cm or more. Both Harmonic Scalpel and LigaSure of 10 mm in diameter, which were used as incision devices, had a length of approximately 35 cm. Therefore, we occasionally needed to pull the stomach toward the tail with forceps of 45 cm in length.

Position where the surgical opening for flexible port insertion is created: forceps, whose rod could be freely controlled at hand with respect to the degree of flexion, allowed the unlimited conduct of surgical procedures. In the case of using straight forceps, it was necessary to establish the site for port setting at a distance of 3 cm or more away from the site of peritoneal reflection toward the mouth in order to avoid the forceps' hit against the pelvis.

Method to operate forceps when using the flexible port: regarding surgical procedures, it was possible to use the flexible port by the same operations of forceps as the cross or parallel method for SPS.

## 4. Discussion

**Issues Related to Ports of Conventional NOTES and to Conventional Techniques**

In conventional NOTES, there has been a risk of intra-abdominal abscess caused by bacterial intrusion from the site of endoscope admittance in the abdominal cavity when no port was used. In the case of using the port for laparoscope-assisted surgery, furthermore, there has been a risk of port fall during surgery because port length was insufficient due to port usage without change and the port surface was slippery. For the application of conventionally existing TEM technologies, an attempt has been made to insert a giant, metal, surgical opening-creating device into the anus and to create and use the transrectal route [4]. This method causes a burden to the anal sphincter and occasionally injures the anus even when using a muscle relaxant. However, the flexible port could be set to the rectum at the surgical opening of several diameters in the GI tract only via the visual field of a colonoscope. Extension of the anus was not required during surgical procedures, which verified the advantage of the flexible port in the aspect of lessening the burden to the anus. Furthermore, there is also an attempt to use as the port for NOTES an overtube port of specified length that can reach the sigmoid colon [5]. However, the attempt has the following problems: air leakage; time is required to close the surgical opening in the rectum; and greater risks of causing stenosis and ruptured suture due to the creation of a large surgical opening in the intestine that is not directly related to surgery.

**Advantages of Using the Flexible Port**

The cuttable flexible port allows its use regardless of anatomical individual differences. Furthermore, the use of the flexible port permits the determination of the port setting position without being influenced by port length. It is necessary to create surgical openings in the GI tract as numerous as required ports. However, we could verify that no air leakage occurred at the site of port insertion because the port was atraumatically set while dilating the pinhole. We confirmed that the use of the flexible port made it possible to use conventional long-type forceps for laparoscope-assisted surgery without change.

**Comparisons of Advantages between the Flexible Port and Other Techniques**

Conventional NOTES has presented technical difficulties and required skill in operations because an endoscope or an endoscope-customized surgical instrument is used to conduct surgery. Furthermore, an endoscope is used to grasp the organ. Therefore, the weakness of bearing power and traction power made is difficult to manipulate the organ without fail. The use of the flexible port allows the conduct of surgery similarly to laparoscope-assisted surgery by using the cross and parallel methods of conventional SPS. Furthermore, the operability of forceps for laparoscope-assisted surgery will further improve if it is a device which has a mechanism to adjust the angle between the handle and the rod or to permit flexion between the rod and the apical structure.

**Challenges Currently Addressed in Relation to the Present Technique**

Forceps with a length of 35 cm or greater is required to conduct surgery of upper abdominal target organs (e.g., stomach and gallbladder) by using the flexible port. Most incision devices currently available have a length of 35 cm. Therefore, it is necessary to use forceps of 45 cm in length in order to pull the stomach toward the tail when using a cutting device for gastrectomy. We are using the currently available forceps of 45 cm in length as the electrosurgical knife. Furthermore, we plan to use a 45 cm rod-like metal stick to displace the intestine or an organ.

**Future Plan in Consideration of the Present Technique**

In the future, we plan to manufacture 40-cm forceps with the bendable apex and a cutting device. A variety of devices for SPS have already been designed and are ready for commercialization. We consider that the flexible port, which permits the conduct of NOTES as a device for SPS, has a potential of revolutionizing NOTES procedures. We will successively report on whether the flexible port is usable for devices for SPS which are to be commercialized later on.

## 5. Conclusion

We considered that the flexible port has a potential of becoming a highly safe port which allows SPS in NOTES by the transrectal and transvaginal routes.

## Figures and Tables

**Table 1:** Results of the study on the efficiency of insertion of various devices for conventional laparoscope through the flexible port.

		Transrectal route									Transrectal vaginal route					
		*∅* 7 mm port			*∅* 12 mm port			*∅* 18 mm port			*∅* 7 mm port			*∅* 12 mm port		

		Insertion	Tmes the device got stuck	Air leakage	Insertion	Tmes the device got stuck	Air leakage	Insertion	Tmes the device got stuck	Air leakage	Insertion	Tmes the device got stuck	Air leakage	Insertion	Tmes the device got stuck	Air leakage

*∅* 5 mm devices	Forceps [Karl dtorz ∗1] [Covidien ∗2] [Novare ∗3]	◯	none	none	◯	none	a little	◯	none	severe	◯	none	none	◯	none	a little
	Endo Clip [Covidien]	◯	none	none	◯	none	a little	◯	none	severe	◯	none	none	◯	none	a little
	Harmonic scalpel [Johnson & Jonson]	◯	2/10	none	◯	none	a little	◯	none	severe	◯	1/10	none	◯	none	a little
	LigaSureAdvance [Covidien]	◯	none	none	◯	none	a little	◯	none	severe	◯	none	none	◯	none	a little
	ENDO CATCH [Covidien]	◯	3/10	none	◯	none	a little	◯	none	a little	◯	1/10	none	◯	none	a little

*∅* 10 mm devices	Endoscope 240i [Olympus]	×	—	—	◯	4/10	none	◯	none	none	×	—	—	◯	2/10	none
	LigaSure [Covidien]	×	—	—	◯	1/10	none	◯	none	a little	×	—	—	◯	1/10	none
	Anvil holder [Karl storz]	×	—	—	◯	none	none	◯	none	a little	×	—	—	◯	none	none
	Stapler i60 [Power Medical]	×	—	—	◯	none	none	◯	none	none	×	—	—	◯	none	none

*∅* 15 mm devices	ENDO CATCH II [Covidien]	×	—	—	×	—	—	◯	2/10	none	×	—	—	×	—	—

∗1 Scissors; Intestinal usage; Maryland, ∗2 Roticulator, ∗3 Real Hand (Maryland).

**Table 2:** Results of the in vivo study. It was possible to perform partial gastrectomy and cholecystectomy by pure NOTES.

	Transrectal route	
	Partial gastrectomy (*n* = 2)	Cholecystectomy (*n* = 2)

The number of port-setting	12 mm (2 routes) 7 mm (1 route)	12 mm (1 route) 7 mm (2 route)

Time between gastric piercing and fully port-setting (min.)	32.5	19.5
Operation time (min.)	187.5	119
Mount of bleeding	<50 g	<50 g
